# Reducing particulate emissions by using advanced engine oil nanoadditives based on molybdenum disulfide and carbon nanotubes

**DOI:** 10.1038/s41598-023-39933-6

**Published:** 2023-08-21

**Authors:** Zuzanna Bojarska, Weronika Goławska, Marta Mazurkiewicz-Pawlicka, Łukasz Makowski

**Affiliations:** Faculty of Chemical and Process Engineering, Warsaw University of Technology, Waryńskiego 1, 00-645 Warsaw, Poland

**Keywords:** Chemical engineering, Nanoscale materials

## Abstract

Nanoadditives can be used to enhance lubricating properties of engine oils. Although many additives have been developed, molybdenum disulfide and carbon nanotubes have attracted significant attention. In this study, we demonstrate that hybrid nanostructures based on these unique materials (MoS_2_/CNTs) positively affect engine oil lubricating properties. Hybrid nanostructures were produced via wet chemical synthesis in impinging jet reactor. This method is characterized by easy scalability and possible continuous operation, which are crucial in material commercialization. The application of 0.5 wt% suspension exhibited the best results, reducing the friction coefficient at the engine operating temperature by up to 26%. Nanoadditives protected the lubricated parts, causing their wear to be considerably lower than the base oil. The effect of nanoadditives on the quality of exhaust gases was also investigated, which has not yet been researched. The application of the oil with MoS_2_/CNT reduced the emissions of solid particles in the gasoline engine exhaust gas. The total volume of particles in the exhaust gas was reduced by 91% and 49% under idling and load-running conditions. This research showed that MoS_2_/CNTs can be successfully used as nanoadditives in engine oils for improving tribological properties, enhancing anti-wear performance, and reducing particle emissions in exhaust gas.

## Introduction

Despite adopting numerous strategies to reduce the use of gasoline-powered vehicles, their usage continues to increase globally. Although there is an awareness of the threat posed by the daily use of cars, the air quality in many areas remains unchanged or have even deteriorated^1,2^. Air pollutant emissions from gasoline engine exhausts mainly include carbon monoxide, nitrogen oxides, volatile organic compounds, and particulate matter. Particulate matter from internal combustion engines consists of elemental carbon, organic compounds, metals, sulfates, nitrates, and other components derived from fuels and lubricating oils in the combustion process and exhaust gas treatment devices. Furthermore, the primary particles form secondary organic aerosols in atmospheric reactions^3^. An increasing number of new technologies, such as electric and hybrid cars, are being developed to reduce car emissions. However, their high price limits their widespread use, particularly considering the continuously changing economic factors^4^. Therefore, developing methods to mitigate the negative effects of the conventionally used vehicles on environment is essential. Engine oils with improved properties can be used to reduce exhaust emissions from conventional combustion engines.

Engine oil plays a significant role in combustion cycles. The primary task of the oil is to cool the engine and ensure an appropriate sealing of the piston rings. In addition, engine oil reduces the friction between moving machine parts, as it creates a thin film that prevents the direct rubbing of these parts. Additionally, it neutralizes the acids formed during fuel combustion and prevents engine corrosion^4,5^. An effective approach to enhance oil properties is the use of additives. Additives are mainly used to reduce the occurrence of friction phenomena, protect the moving parts of machines against wear, and reduce pollution caused by emitted gases. Additionally, the engine oil plays a crucial role in cooling the engine, leading to lower local oil temperature gradients when additives are utilized. This improvement results not only from lower friction in the system but also from enhanced thermal conductivity. This helps maintain the oil's quality and performance, thereby extending its useful life and reducing the frequency of oil changes. The development of new additives for engine oils with excellent rheological properties, low friction, surface geometry, and low manufacturing costs will facilitate the production of more durable internal combustion engines with increased efficiency than the existing^6–8^.

The use of nanoadditives represents a promising and innovative strategy aimed at significantly enhancing the properties and performance of engine oils. Nanoadditives, owing to their size at the nanoscale level, offer distinct advantages over conventionally used additives, such as ZDDP (Zinc Dialkyl Dithiophosphate). One of the key advantages of nanoadditives lies in their ability to achieve superior dispersion throughout the engine oil. Due to their small dimensions, nanoadditives disperse more uniformly, ensuring that they are effectively distributed to all critical parts of the engine, even in areas that were previously challenging to reach^9–11^. Nanolubricants also help mitigate the environmental issues associated with the use of conventional additives containing sulfur, chlorine, and phosphorus^12^. Owing to their extraordinary mechanical properties and good chemical stability, carbon nanotubes (CNTs) have gained significant research interest for improving the tribological properties of engine oils^7,13,14^. CNTs can self-assemble in lubricating zones owing to their unique morphology. Therefore, the sliding contact area, shear stress, friction, and wear can be reduced^13^. Molybdenum disulfide (MoS_2_) nanoparticles are also promising nanoadditives for engine oils. They consist of individual layers of covalently linked S-Mo-S, and weak van der Waals interactions exist between these layers, ensuring a low friction coefficient^15–17^. A highly promising approach for enhancing oil lubricating properties is to use the synergistic combination of different nanoadditives. This novel strategy harnesses the advantages of both materials, resulting in improved dispersion, reduced friction, and increased engine longevity. Additionally, the integration of nanomaterials addresses environmental concerns, making it a key contender in the future of lubrication technology. Song et al. synthesized a composite based on MoS_2_ grown on CNTs via the chemical vapor deposition method. They established that dibutyl phthalate (DBP)-containing strongly oxidized CNTs and molybdenum disulfide (SOCNTs@MoS_2_) composites exhibited the highest tribological and antiwear performance among DBP additives, such as SOCNTs, MoS_2_, and SOCNT-MoS_2_ mixtures^18^. Zhang et al. also confirmed that hybrid nanoparticles have better tribological performance than pure MoS_2_ or CNTs during the minimum quantity lubrication grinding of Ni-based alloys^19^.

In our previous study, we precipitated MoS_2_ nanoparticles on various carbon nanomaterial surfaces using a facile and scalable method^5,9,20^. The obtained hybrid nanostructures, including MoS_2_/CNTs, were tested as engine oil nanoadditives. The synergetic effect between MoS_2_ and CNTs enabled the production of engine oil with enhanced tribological properties compared to the base oil or that modified with pure MoS_2_. This improvement was due to the smaller size of the MoS_2_ particles precipitated on the carbon surface, better dispersion, outstanding lubricating properties of MoS_2,_ and excellent mechanical and thermal strengths of the CNTs^5^.

In this study, we synthesized hybrid nanostructures based on MoS_2_ and CNTs using wet chemical synthesis in an impinging jet reactor. Various concentrations of MoS_2_/CNTs in typical engine oil were tested to achieve the optimal tribological properties. The effect of the nanoadditives on the wear of the lubricating parts was also investigated. Moreover, the influence of the oil used on the particle distribution in the engine exhaust gases was investigated. Owing to the aforementioned environmental issues, these studies are essential for obtaining engine oils with enhanced properties. To the best of our knowledge, this is the first study to investigate the effect of hybrid nanostructures based on MoS_2_ and CNT nanoadditives on the quality of exhaust fumes.

## Materials and methods

### MoS_2_/CNT hybrid nanostructures

Hybrid nanostructures based on molybdenum disulfide and multiwalled CNTs, MoS_2_/CNTs, were synthesized via wet chemical precipitation in an impinging jet reactor, as described in Refs.^5,20^. First, 8.828 g of ammonium heptamolybdate tetrahydrate (extra pure powder, Merck Millipore, United States) was added to a small amount of deionized (DI) water (~ 100 mL) in a 250 ml volumetric flask. Next, 19.212 g of citric acid (≥ 99.5%, Sigma-Aldrich, United States) was added gradually. The mixture was then stirred at 90 °C until a clear solution was obtained. To this solution, 1.067 g of multiwalled CNTs and DI water was added until the total volume was 250 ml. Multiwalled CNTs were grown via chemical vapor deposition with a purity of at least 95 wt% (CNT Co., LTD., South Korea). The CNTs were dispersed in an ultrasonic bath for 30 min to expose the carbon surface. In another 250 ml volumetric flask, 34 ml of 20 wt% ammonium sulfide (Sigma-Aldrich, United States) was diluted with DI water. The reaction was performed in an impinging jet reactor maintained at 20 °C. The precipitate was further purified via centrifugation and heat treatment at 550 °C under an inert gas flow.

### Material characterization

As particle size is a crucial factor determining additive properties, which influence the stability and tribological properties of the lubricant, the particle size distribution (PSD) of MoS_2_/CNTs was determined using a laser diffraction particle size analyzer LS 13 320 (Beckman Coulter Life Sciences, United States). The specific size of the purified product (arithmetic average, d_10_) was calculated using the method of moments^5^. To estimate the mass ratio of MoS_2_ to CNTs, thermogravimetric analysis (TGA) of the hybrid nanostructures was conducted using a TGA/DSC 3 + analyzer (Mettler Toledo, Switzerland). The material was heated from 30 to 1200 °C at a heating rate of 10 °C/min in a 60 ml/min airflow. The morphology and structure of the MoS_2_/CNTs were analyzed via scanning electron microscopy (SEM) and X-ray powder diffraction analysis (XRD). A small amount of MoS_2_/CNTs was dispersed in isopropanol. Thereafter, a few drops of the suspension were applied to the transmission electron microscopy (TEM) copper grid with a carbon film. The images were captured in two modes: secondary electron (SE) and bright-field scanning transmission electron microscopy (BF-STEM) using a field-emission scanning electron microscope S5500 (Hitachi, Japan) with a resolution of 0.4 nm. The XRD measurements were conducted using a D8 Advance diffractometer equipped with a copper lamp (40 kV, 40 mA) and a LynxEye detector, following the Bragg–Brentano configuration (Brucker, United States).

### Oil-based suspensions

Hybrid nanostructures of MoS_2_/CNTs were used as oil additives. Various suspension concentrations were tested (i.e., 0.05; 0.1; 0.2; 0.4; 0.5; 0.6; 0.7; 0.8; 0.9; 1.0 wt%). For this study, one of the most common multigrade 10W-40 engine oil (Liqui Moly, Germany) was used as the base oil.

### Tribological performance

The tribological performance of lubricants is another crucial factor affecting the oil properties. Five milliliters of the obtained suspensions (from Section “Oil-based suspensions”) were dispersed and degassed using an ultrasonic homogenizer UP400S (Hielscher, Germany) for 15 min in a cooling bath. The tribological properties of the suspensions and base oil were analyzed using an MCR tribometer (T-PTD 200) with a ball-on-three-pin setup (Anton Paar, Austria)^5^. A new set of balls and pins was used for each sample; 100Cr6 steel pins and balls were purchased from Anton Paar. The engine can produce the most emissions during the first few minutes after starting due to the required rich air–fuel mixture in cold engines and the inefficient catalytic converter under cold conditions. Due to the changing weather throughout the year, the measurements were performed at − 10, 0, 25, 75, and 100 °C. The measurements for each suspension were repeated three times.

### Rheological performance

Owing to the significance of rheological properties of engine oils for lubricating applications, it is crucial to determine the effects of additives on the rheological properties of the oil. Therefore, twenty-five milliliters of the optimal suspension obtained in this study was dispersed and degassed using an ultrasonic homogenizer for 25 min. The rheological properties of the suspensions and base oil were measured using an MCR 302 rheometer (Anton Paar, Austria) with a CC28.7 measuring cylinder and CC27 measuring cup^5^. The measurements were performed at − 10, 0, 25, 75, and 100 °C. The measurements for each suspension were repeated three times. Based on the results, the consistency index ($k$) and flow behavior index ($n$) of the Ostwald-de Waele model for the tested oils were determined. The obtained rheograms were corrected in the region of high shear rates using the model of non-isothermal Couette flow, as described in Ref.^9^.

### Suspension stability

To evaluate the suspension stability, the friction coefficient of the optimal suspensions and base oil was measured over time. The tests were performed at a temperature of 75 °C and a constant sliding speed of 0.1 m/s. In addition, the influence of the normal force on the friction coefficient was determined. The measurement lasted for 30 min for each value of normal force. The amount of the tested lubricant and the measuring system was the same as that in Section “Tribological performance”.

### Wear measurements

Wear is an important aspect in tribology. The optimal suspension and base oil friction coefficients were measured for approximately 3 h. Tests were performed for a sliding velocity of 0.1 m/s at 75 °C. After the measurements, images of the pin scratches were captured using a Delta Optical Discovery microscope. The amount of the tested lubricant and the measuring system was the same as that in Section “Tribological performance”.

### PSD of engine exhaust

Oil additives are used to lower the friction coefficient, protect moving parts, and reduce pollution due to engine exhaust. Therefore, the PSDs in the exhaust gas of a gasoline engine filled with the base oil and optimal suspension obtained in this study were analyzed. First, 400 ml of the suspension was dispersed using an ultrasonic homogenizer for 45 min in a cooling bath. Measurements were performed using fast aerosol particle emission spectrometry—FAPES (Grimm Aerosol Technic GmbH system, Germany)^21^. The exhaust gases were produced using a 5.5 kW EM 5500CX Honda (Japan) power generator for generating 230 V and 12 V alternating current. The device has an economical four-stroke internal combustion engine 389 cc Honda GX390 air-cooled. Its net power output is 1.7 HP at 3,600 RPM. The engine was additionally air-cooled by a fan. The produced flue gases were then fed to the combustion chamber, where the measuring probe was located. The test started by determining the background (engine off) for 3 min. The PSD was estimated during the idle operation of the engine for 3 min; thereafter, the load was turned on for another 2 min, and the measurements were continued. The load was an electric heater with a capacity of 2 kW, which was set to full power. The same fuel obtained from the Polish oil company ORLEN was used to perform a series of measurements. In addition, before pouring oil into the power generator, the inside of the engine was washed with base oil in order to get rid of any impurities and particles from previous tests. The measurements were conducted in the presence of a CO detector. Additionally, work safety was ensured by removing exhaust fumes from both the combustion chamber and laboratory room using fans.

## Results and discussion

### Material characterization

**Figure 1:**
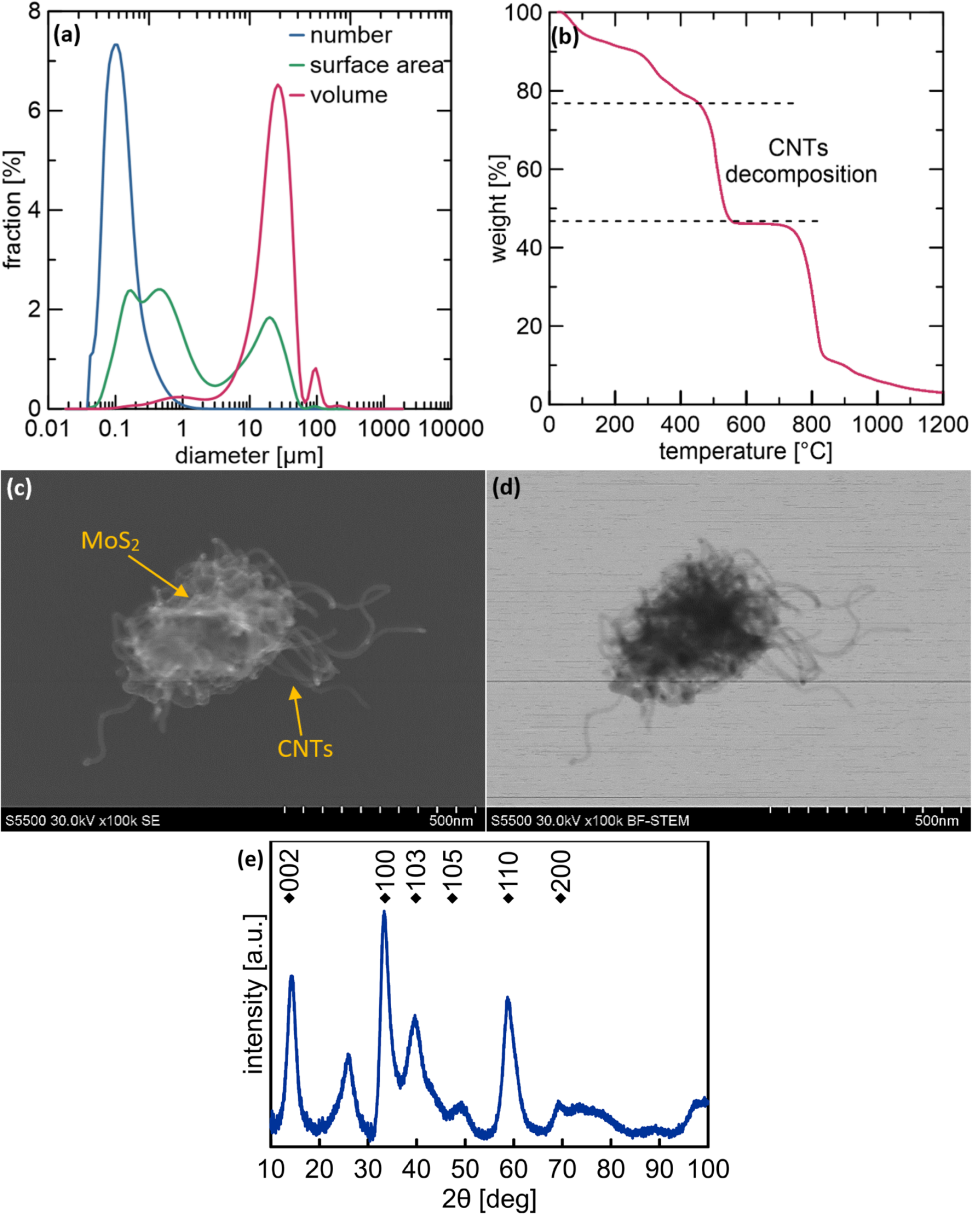
(**a**) PSDs by number, surface area, and volume, (**b**) TGA, (**c**) SEM images in the SE and (**d**) BF-STEM modes, (**e**) diffractogram of MoS_2_/CNTs.

The specific size d_10_ was calculated based on PSD (Fig. 1a). The mean size of the MoS_2_/CNT hybrid nanostructure was 0.146 µm. The analysis of the particle volume PSD revealed the presence of agglomerates, which may have originated because of either the geometry of the CNTs or the formation of larger forms during annealing. However, agglomerates were not visible in the PSD by number, indicating a negligible amount. In a previous study, we determined the CNT content in samples based on the support decomposition temperature^22^. The mass loss corresponding to the CNT content of the sample was 30% (Fig. 1b). The SEM images showed that the MoS_2_ deposited on the CNTs stuck them together and caused the formation of spherical forms (Fig. 1c,d). XRD is a widely used technique for determining the crystal structure of materials (Fig. 1e). In this case, the observed peaks corresponded to the 2H phase of MoS_2_, which is the most stable and commonly observed phase. Additionally, the average size of the MoS_2_ crystallites was found to be in the range of 2–3 nm. Owing to the weak van der Waals interactions between MoS_2_ layers, these forms can be easily exfoliated to smaller sizes during lubrication. In addition, the decomposition of the carbon support was not observed during annealing. Therefore, both MoS_2_ and CNTs may influence tribological properties.

### Tribological performance

**Figure 2:**
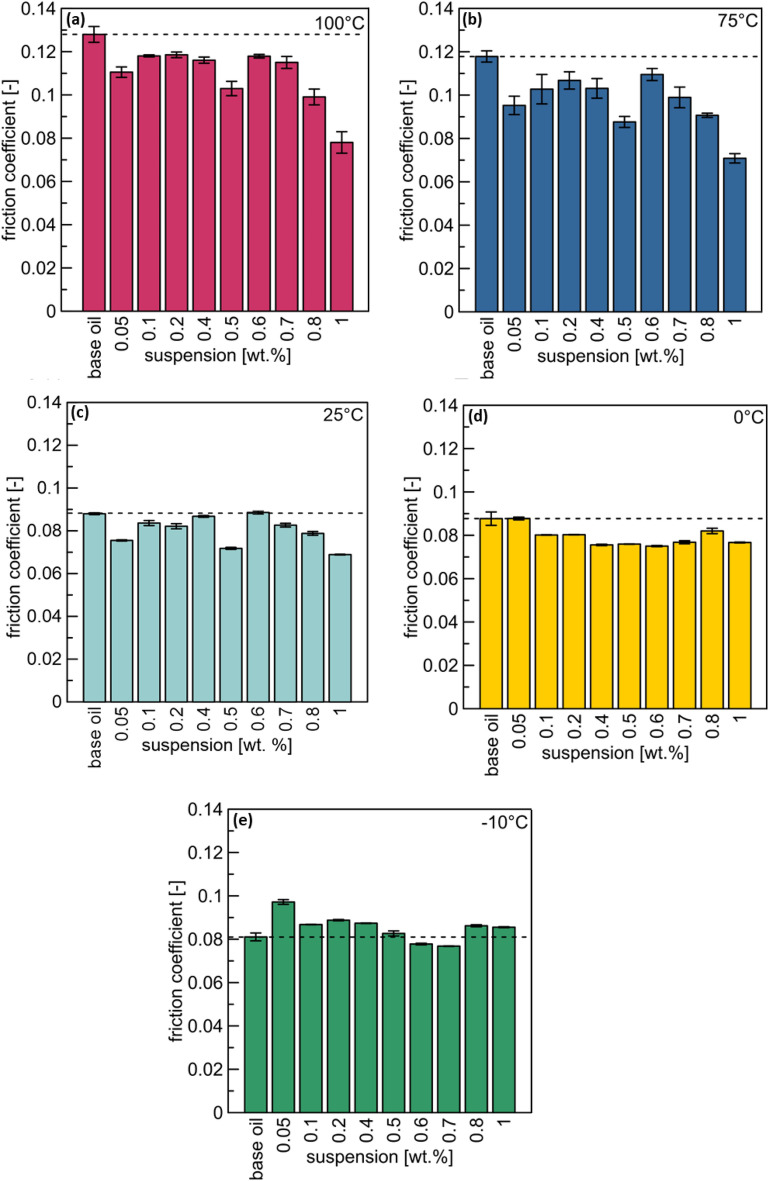
Friction coefficient at a sliding velocity of 0.63 m/s for each suspension and base oil at (**a**) 100, (**b**) 75, (**c**) 25, (**d**) 0, and (**e**) − 10 °C.

**Figure 3:**
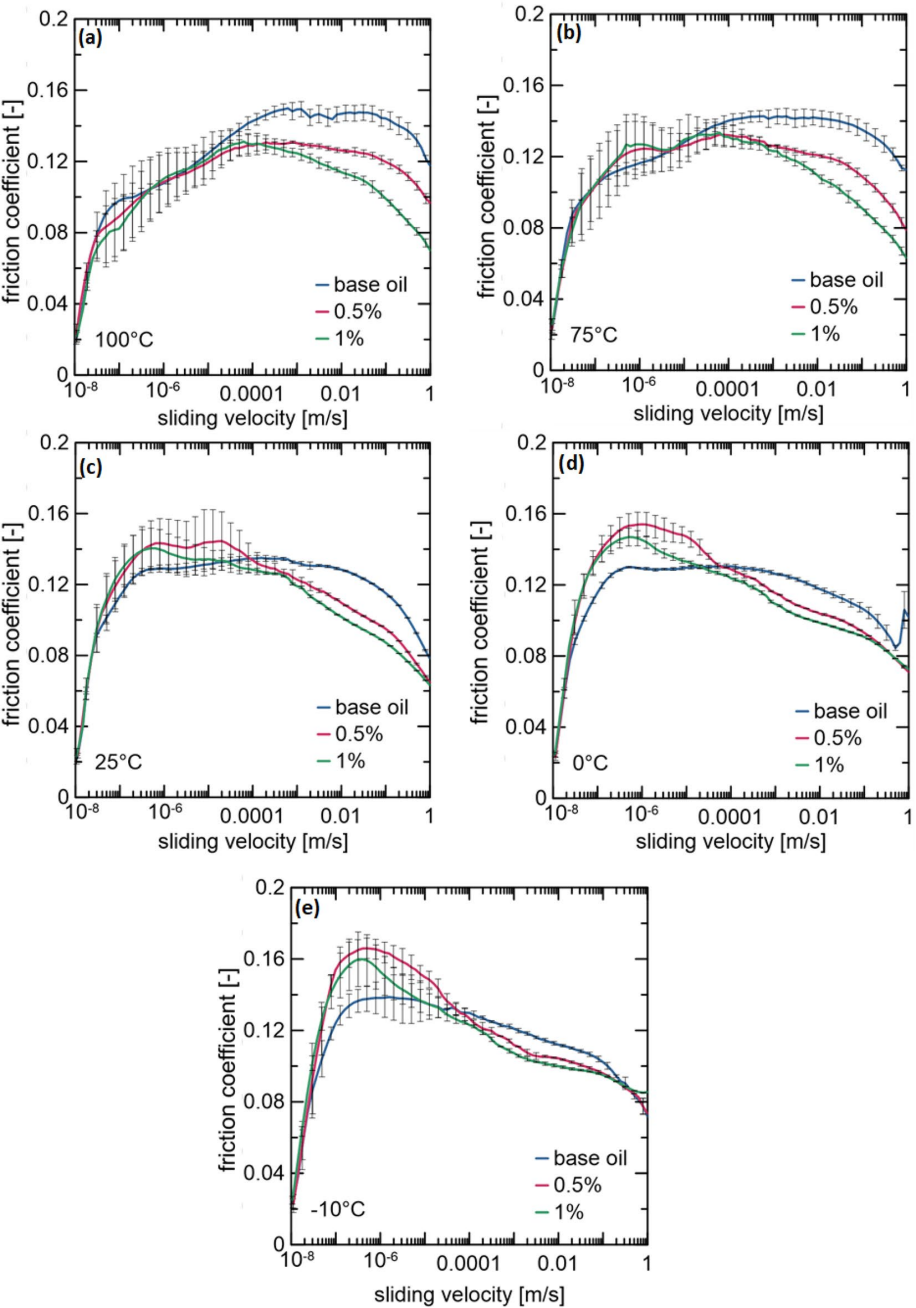
Modified Stribeck curves for 0.5 and 1 wt% suspensions and the base oil at (**a**) 100, (**b**) 75, (**c**) 25, (**d**) 0, and (**e**) − 10 °C.

Modified Stribeck curves showing the relationship between the friction coefficient and sliding velocity were obtained for the base oil and each suspension at various temperatures (-10, 0, 25, 75, and 100 °C). Considering the chart readability, the dependence of the friction coefficient has been presented for a representative sliding velocity, 0.63 m/s (Fig. 2). At temperatures close to the engine operating temperatures (75 and 100 °C), the improvement in tribological properties of each suspension is superior to that of the base oil (Fig. 2a,b). The 0.5 and 1 wt% MoS_2_/CNTs exhibited the highest performance. The lowest friction coefficient values for the sliding velocity of 0.63 m/s were obtained for the 1 wt% suspension. A decrease of 40% and 39% in the values compared to those of the base oil were observed at 75 and 100 °C, respectively. For 0.5 wt% suspension, the decrease in values was 26% and 20% at 75 and 100 °C, respectively. At 25 °C (Fig. 2c), no significant differences were observed between the two suspensions. However, the hybrid nanostructure deteriorates the tribological properties at lower temperatures (Fig. 2c,d). At these temperatures, the rheology of the oil increases significantly, which may result in a change in the mobility of the additives in the suspension. The relationship between the tested friction coefficient and the additive content is not linear and can be influenced by many factors. First, the results shown correspond to only a segment of the entire modified Stribeck curve. Moreover, an optimum relationship exists between the addition amount of the friction-reducing agent and its tendency to agglomerate and cause further sedimentation. Considering different values of the sliding velocity, 1 wt% and 0.5 wt% were selected as the optimal concentrations, and their modified Stribeck curves with the base oil are presented in Fig. 3.

Based on the modified Stribeck curves (Fig. 3), the optimal tribological performance was exhibited by a suspension with a concentration of 1 wt%, reducing the friction coefficient of the base oil up to 39% and 40% at 100 and 75 °C, respectively. Additionally, the use of the 0.5 wt% suspension decreased the friction coefficient values up to 20% and 26% for temperatures of 100 and 75 °C, respectively. However, the enhancement in the tribological properties was visible only for high sliding velocities (≥ 0.0001 m/s). At low values, the oils behaved similarly. Tests conducted at 25 °C (Fig. 3c) showed similar changes in the tribological properties of the suspensions. At low sliding velocities, the deterioration of the tribological properties can be observed. However, these velocities are irrelevant for application in car engines. Contrary to the case of the high temperatures, wherein hydrodynamic lubrication exists, at 0 and − 10 °C, oils operate in the mixed lubrication regime (Fig. 3d,e). As in the case of 25 °C, for low values of the sliding velocity (< 0.0001 m/s), the suspensions exhibited higher friction coefficients than the base oil. For high values, both suspensions were characterized by lower friction coefficient than that of the base oil. At these temperatures, the reduced nanoadditive impact was related to higher viscosity. In summary, the 1 wt % suspension delivered the optimal performance during the short time of measuring the modified Stribeck curve.

### Rheological performance

**Figure 4:**
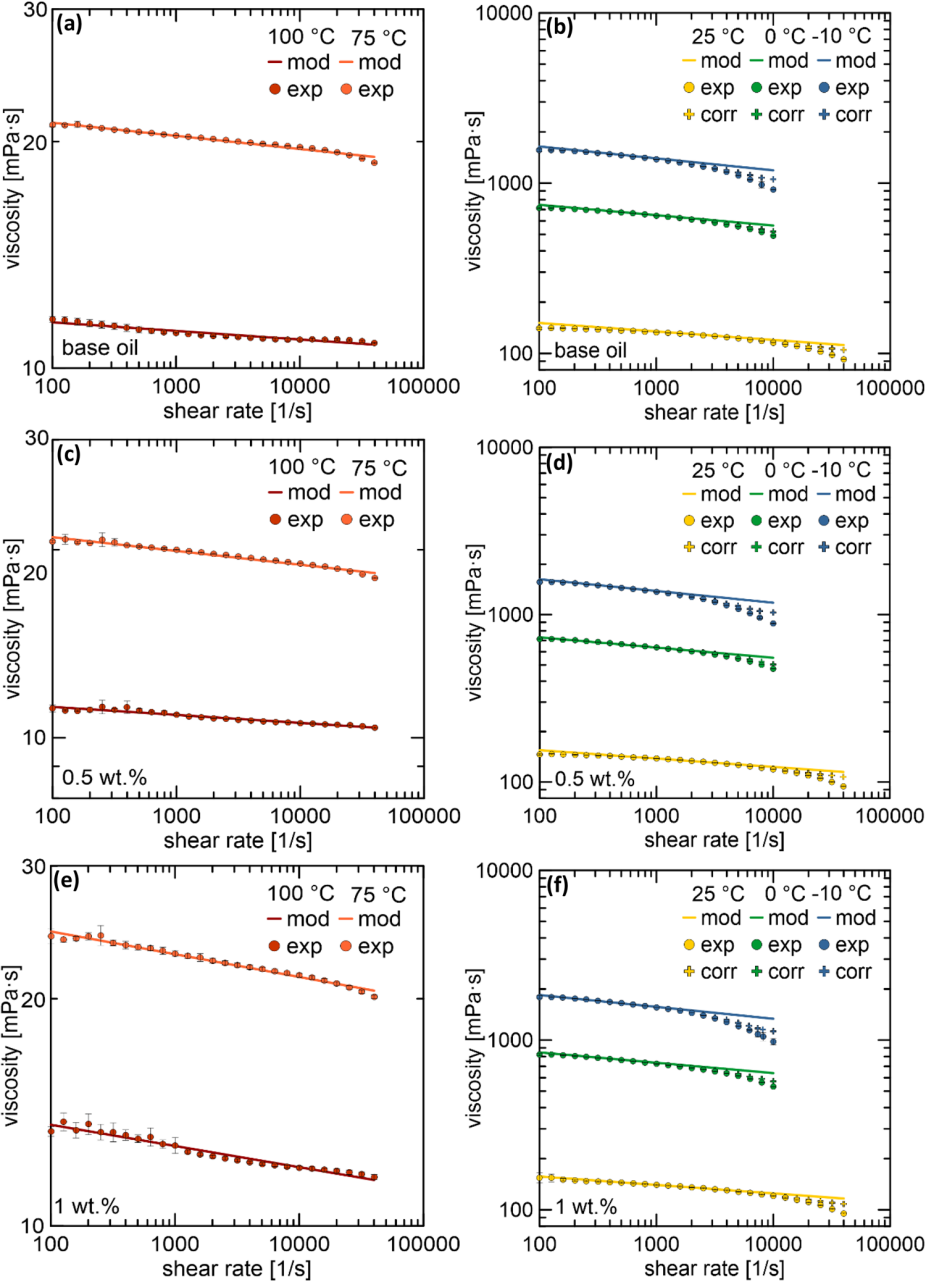
Rheograms for (**a,b**) base oil and (**c,d**) 0.5 and (**e,f**) 1 wt% suspensions at 100, 75, 25, 0, and − 10 °C (mod—Ostwald-de Waele model, exp—experiment, corr—corrected using the non-isothermal Couette flow model).

Considering the defined rheology of engine oils, it is essential to determine the effect of additives on the viscosity of the base oil. The 0.5 wt% suspension and base oil rheograms showed no significant difference (Fig. 4) at all temperatures. The most significant variation was observed at 25 °C at a low shear rate value of 4%. For the 1 wt% suspension, an increase in viscosity was noticeable at all temperatures. The increased viscosity of the oil may result in the formation of a thicker film between the moving parts, resulting in better lubricating properties, confirming the tribological results. However, the increased viscosity worsens the hydrodynamic properties of the oil, which are essential because of the oil pump working conditions.

The $k$ and $n$ values according to the Ostwald-de Waele model (Eq. (1)) were determined (Table 1). For temperatures lower than 25 °C and in the region of high shear rates, where the viscous heating effects are the most significant, the non-isothermal Couette flow model was applied to correct the rheograms. The exact method for determining the temperature correction is described in Ref.^9^.

**Table 1:** Consistency and flow behavior indexes for the base oil and suspensions.

	T [°C]	T [K]	n [−]	k [Pa·s^n^]
Base oil	− 10	263.15	0.930	2.263
	0	273.15	0.939	0.989
	25	298.15	0.950	0.190
	75	348.15	0.983	0.023
	100	373.15	0.989	0.012
0.5 wt%	− 10	263.15	0.930	2.550
	0	273.15	0.939	1.120
	25	298.15	0.950	0.198
	75	348.15	0.978	0.023
	100	373.15	0.987	0.012
1 wt%	− 10	263.15	0.930	2.425
	0	273.15	0.939	0.970
	25	298.15	0.950	0.195
	75	348.15	0.970	0.028
	100	373.15	0.972	0.016

1 $$\tau =k{\dot{\gamma }}^{n}$$

### Suspension stability

**Figure 5:**
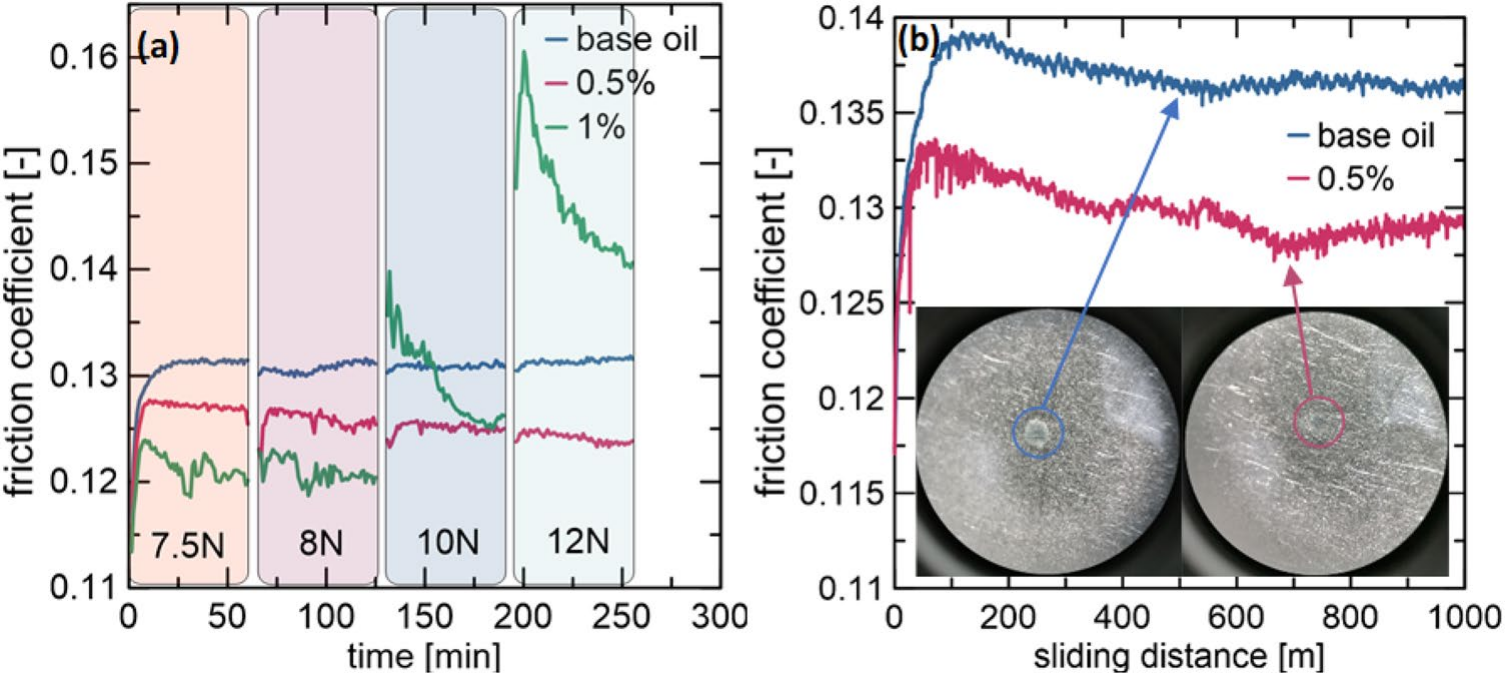
Friction coefficient over time with changing normal force for the base oil and 0.5 and 1 wt% suspensions (**a**); wear measurements for base oil and 0.5 wt% suspension (**b**).

In a previous study^5^, the stability of 1 wt% suspensions with the addition of MoS_2_ and MoS_2_ deposited on various carbon nanomaterials was studied. We demonstrated that the MoS_2_ precipitated on the carbon material possessed more petite sizes than pure MoS_2_. In addition, the overall size of the hybrid particles was smaller than that of the pure MoS_2_ produced via the same method. Owing to the smaller size, more developed surface, and lower density of hybrid particles, the suspensions showed better dispersion and stability than those with pure MoS_2_. However, over time, a precipitate formed at the bottom of each suspension. Therefore, stability tests were performed using a tribometer. The friction coefficient measured over time is shown in Fig. 5a. In addition, the normal force used was changed during the test to assess the lubricant behavior under changing conditions. No significant difference in the friction coefficient was observed for the base oil over time or with varying normal forces. The 1 wt% suspension exhibited the optimal performance for more than two hours of the test. However, increases in time and normal force significantly changed the tribological performance of the suspension. At high concentrations, particle agglomeration is more likely to occur over time. Moreover, an increase in the normal force reduces the thickness of the lubrication film. Therefore, agglomerated particles can deteriorate the tribological properties of the suspension. However, the addition of 0.5 wt % MoS_2_/CNTs lowered the friction coefficient, with increasing time and normal force, demonstrating the optimal tribological properties and good suspension stability.

### Wear measurements

The anti-wear performance of the MoS_2_/CNTs nanoadditive was evaluated. Figure 5b presents the friction coefficient as a function of the sliding distance for the base oil and suspension. A decrease in the friction coefficient during the measurement was noticeable for both lubricants. The run-in times for the 0.5 wt% suspension was shorter than those for base oil. However, initially, abrupt decreases in the friction coefficient were observed, which most probably corresponded to the exfoliation of the particles owing to the shear stress during the sliding of the friction surfaces. The suspension performed better for the entire sliding distance range than the base oil. The pin scratches that appeared after the measurements are also presented in Fig. 5b. These scratches were barely visible and smaller than those on a pin lubricated with the base oil. During abrasion, the additives created a thin protective film on the pin surface. Hence, the surface of the pin was less-scratched.

### Friction and wear mechanisms

The large surface area of CNTs allowed the formation of hybrid nanostructures with a smaller particle size than that of pure MoS_2_ obtained using the same method, as confirmed in Ref.^5^. This prevents particle agglomeration, resulting in better dispersion and tribological performance than those of unmodified nanoadditives or MoS_2_-CNT mixtures.

Based on the obtained results, two aspects should be considered: (i) the lubrication mechanism of the hybrid MoS_2_/CNT nanostructure and (ii) influence of the concentration of the nanoadditive on the tribological properties of the engine oil.

**Figure 6:**
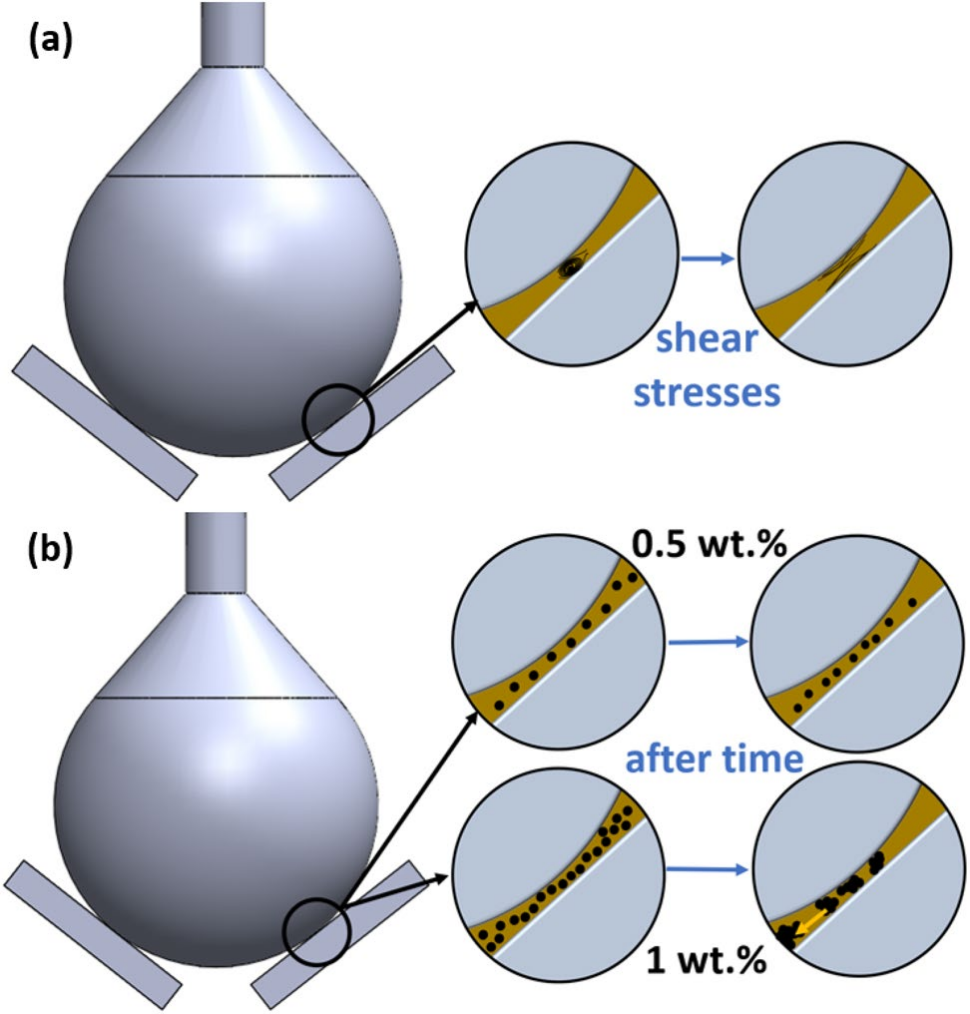
(**a**) Predicted exfoliation of the MoS_2_/CNT nanoadditives due to shear stress during lubrication; (**b**) predicted agglomeration and sedimentation of the MoS_2_/CNT nanoadditves over time.

(i) The tested oils were shear-thinned. The nanoadditives positively affected the oil tribological performance at lower oil viscosities, that is, higher temperatures and sliding velocity values. This may be due to the exfoliation of the hybrid particles because of shear stresses during abrasion. A similar dependence was observed in a previous study^23^. Although the particles initially had a spherical shape, their structure might have changed entirely because of grinding. Owing to shear stress, the fragmented particles can adopt forms that are reminiscent of nanosheets, in which the tribological properties are influenced by both CNTs and MoS_2_. The exfoliated particles may also have a good accessible contact with the lubricated surface, creating a protective film. Therefore, during the stability and wear tests, the friction coefficient decreased. The ideological scheme of this process is illustrated in Fig. 6a. In addition, certain materials may adhere to the abrasive surface, thereby protecting this surface from abrasion. Therefore, scratches on the pins after the wear measurements were smaller for the suspension than those for the base oil. At higher viscosities, that is, lower temperatures and sliding velocities, a thicker hydrodynamic film was observed between the moving parts. In addition, the particles may not be exfoliated during lubrication because of their low mobility in oil, which could have negatively affected the tribological properties.
(ii) The concentration also influences the tribological properties of the suspensions. The rate of agglomeration is higher at higher concentrations. In the case of agglomerates, the sedimentation tendency is considerably higher than that of primary particles. The system used in this study consisted of pins and a ball placed at an angle of 45° to each other. Therefore, agglomeration and sedimentation were observed during the measurements. Stability tests showed that the 1 wt% suspension particles began to agglomerate and sediment over time. An increase in normal force reduced the thickness of the lubricating film, and the presence of agglomerates deteriorated the tribological properties. In the case of the 0.5 wt% suspension, no similar phenomenon was observed. Hence, it can be considered that this content resulted in a stable suspension with the desired lubricating properties. The ideological scheme of this process is presented in Fig. 6b.

### PSD of engine exhaust

**Figure 7:**
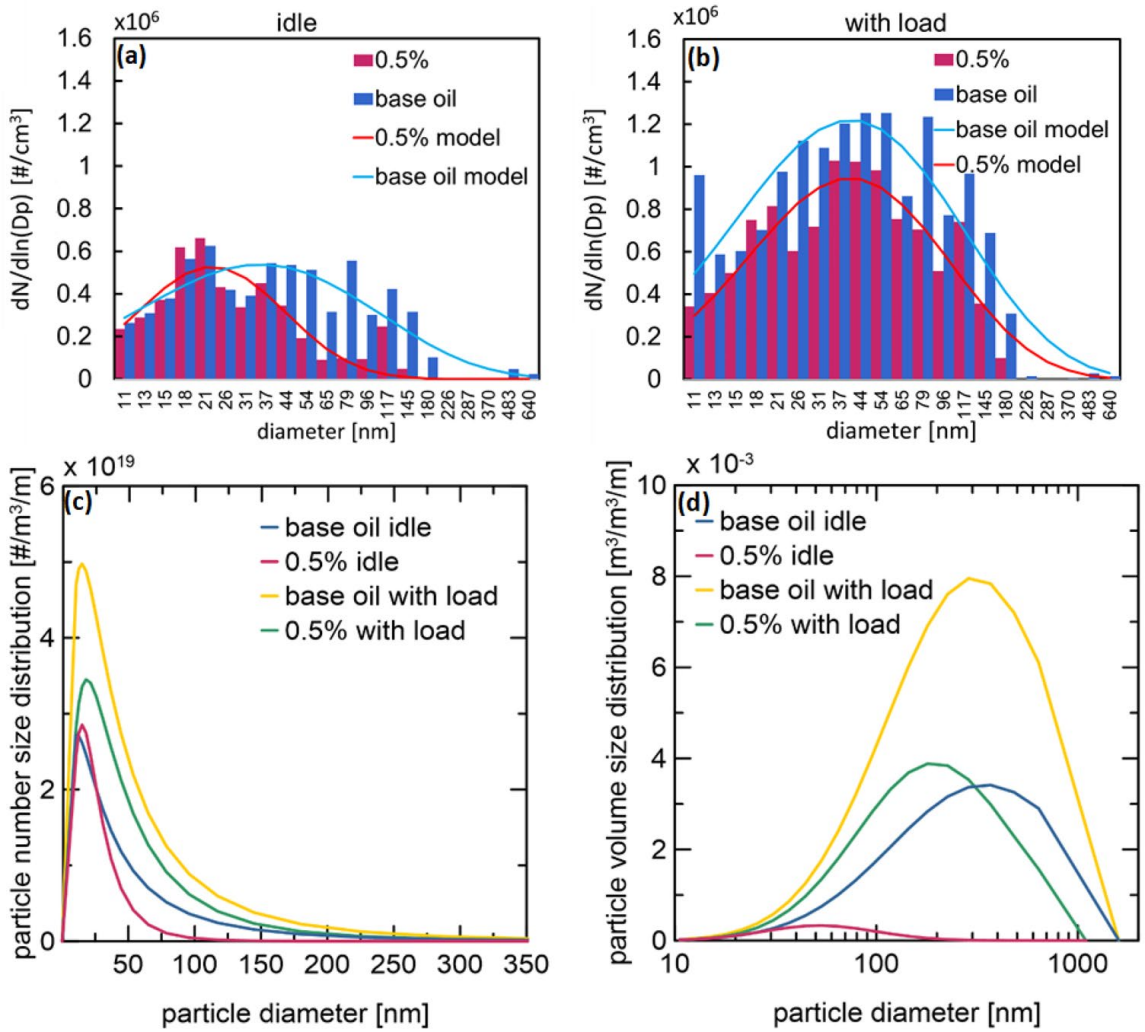
Normalized PSD measured using FAPES (**a**) at the idle run and (**b**) with load, (**c**) particle number size distribution, and (**d**) particle volume size distribution of engine exhaust.

An advantage of using oils with additives is the reduction in exhaust emissions. Therefore, the PSD of the exhaust gases of the engines operating with the base oil and 0.5 wt % suspension under idle and load conditions was investigated. Based on the PSDs obtained using FAPES (Fig. 7a,b—histograms), the normal distribution was expressed as a function of the logarithm of particle size (Figs. 7a,b—solid lines). Furthermore, the results were converted into PSDs by number (Fig. 7c) and volume (Fig. 7d) using Eqs. (2), (3), (4) and (5), assuming that the volume shape factor of the particles is equal to $\frac{\pi }{6}$^24^. The primary particles measured using FAPES had a spherical shape, and fractal-like particles were formed only after deposition on the surface.

2 $$\mathrm{d\,ln}\,{D}_{p}=\frac{\mathrm{d\,ln}\,{D}_{p}}{\mathrm{d}{D}_{p}}\cdot \mathrm{d}{D}_{p}=\frac{\mathrm{d}{D}_{p}}{{D}_{p}}$$

3 $$\frac{\mathrm{d}N}{\mathrm{d\, ln}\left({D}_{p}\right)}=\frac{\mathrm{d}N}{\mathrm{d}{D}_{p}}{D}_{p}$$

4 $$\frac{\mathrm{d}N}{\mathrm{d}{D}_{p}}=\frac{\mathrm{d}N}{\mathrm{d\, ln}\left({D}_{p}\right)}\frac{1}{{D}_{p}}$$

5 $$\frac{\mathrm{d}V}{\mathrm{d}{D}_{p}}=\frac{\mathrm{d}N}{\mathrm{d}{D}_{p}}\frac{\pi }{6}{{D}_{p}}^{3}$$

The results confirmed that the nanoadditives positively affected the quality of engine exhaust, which results from the smaller intensity of the normal distributions’ curves obtained for the nanosuspension than the base oil. Moreover, the number and total volume of particles contained in the exhaust gases were significantly smaller when the engine worked with 0.5 wt% suspension than those with the base oil. This effect is visible for operation under both load and idling conditions. The total volume of particles in the exhaust gas was reduced by 91% during idling and 49% during the load running. The addition of MoS_2_/CNTs to the base oil improved its lubricating and cooling properties, contributing to a reduction in oil consumption and the nucleation of nanoparticles formed during engine operation.

## Conclusions

In this study, hybrid nanostructures of MoS_2_/CNTs prepared via wet chemical synthesis in an impinging jet reactor were tested as engine oil additives. Oils with added MoS_2_/CNTs showed improved tribological properties, particularly at high temperatures, close to the operating conditions of a car engine. The optimal concentrations of the oil suspension were determined to be 0.5 and 1 wt%, and these suspensions reduced the value of the friction coefficient to 26% and 40%, respectively. However, in long-term studies, the agglomeration of particles was observed in the 1 wt% suspension, while the 0.5 wt% suspension maintained and even improved its properties over time. The anti-wear properties of the MoS_2_/CNT oil nanoadditive were also investigated. A lubrication mechanism was proposed in which particle exfoliation during the lubrication of rubbing surfaces was considered. Furthermore, the addition of 0.5 wt% suspension did not significantly affect the rheological behavior of the oil as in the case of 1 wt%. The Ostwald-de Waale model was used to describe the rheology of the obtained oil suspensions. The effects of nanoadditives on particulate emissions during the operation of a gasoline engine were also investigated. For the 0.5 wt% suspension, the total volume of particles in the exhaust gas was reduced by 91% and 49% under idling and load-running conditions. This study demonstrates that MoS_2_/CNT hybrid nanostructures produced via a simple and scalable method can be successfully used as nanoadditives for engine oils to improve their lubricating properties.

## Data Availability

The raw/processed data required to reproduce these findings cannot be shared at this time due to technical or time limitations, but are available from the corresponding author on reasonable request. All authors declare that they have no conflicts of interest.
